# Supplementary material for: A reconciled solution of Meltwater Pulse 1A sources using sea-level fingerprinting
Source: Nat Commun. 2021 Apr 1;12:2015. doi: 10.1038/s41467-021-21990-y (PMC8016857; doi:10.1038/s41467-021-21990-y)
Supplement: Supplementary file 1 — Supplementary Information [file 41467_2021_21990_MOESM1_ESM.pdf]

Supplementary Information for: A reconciled solution of Meltwater Pulse 1A sources using sea-level fingerprinting

Yucheng Lin[1,3*], Fiona D. Hibbert[2,3], Pippa L. Whitehouse[1], Sarah A. Woodroffe[1], Anthony Purcell[3], Ian Shennan[1], and Sarah L. Bradley[4]

[1]Department of Geography, Durham University, Durham, DH1 3LE, UK
[2]Department of Environment and Geography, University of York, York, YO10 5NG, UK
[3]Research School of Earth Sciences, Australian National University, Canberra, ACT 0200, Australia
[4]Department of Geography, The University of Sheffield, Sheffield, S10 2TN, UK

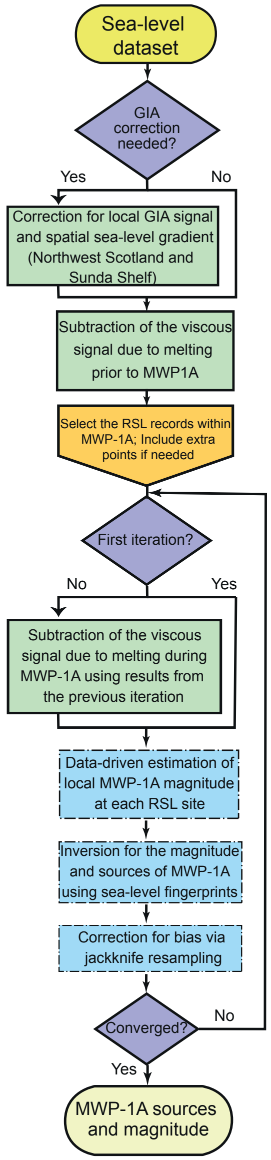

Supplementary Fig. 1: Flow chart of the iterative inversion procedure adopted in this study. Green boxes denote steps where GIA modelling is used to isolate the fingerprint signal, blue boxes represent steps in the data-driven statistical inversion, and purple boxes are conditional statements.

*yucheng.lin@durham.ac.uk

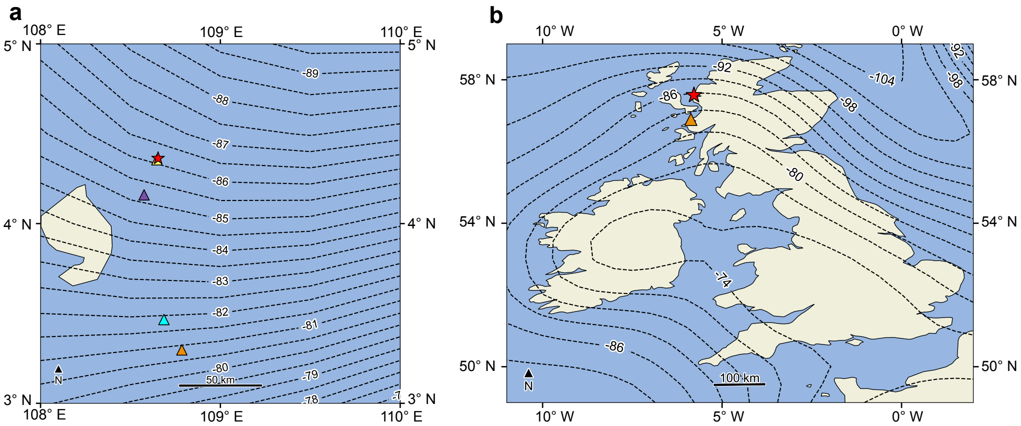

Supplementary Fig. 2: Spatial distribution of RSL across (a) Sunda Shelf (at 14.5 ka BP) and (b) Northwest Scotland (at 14.53 ka BP) due to far-field ice loss. The contour lines are determined from the mean of 240 GIA models. Red stars denote the location of site 18300 (Sunda Shelf) and site Applecross (Northwest Scotland), to which all other sites are referenced when correcting for the sea-level spatial gradient. Triangles with different colours are the different coring sites, Sunda Shelf: site 18301 (yellow), site 18302 (purple), site 18309 (cyan) and site 18308 (orange); Northwest Scotland: Arisaig (orange). Colours correspond to those used in main text Fig. 2c-f. The RSL prediction shown in (b) is derived using the global ice models of ICE6G_C and ANU without the British-Irish Ice Sheet component.

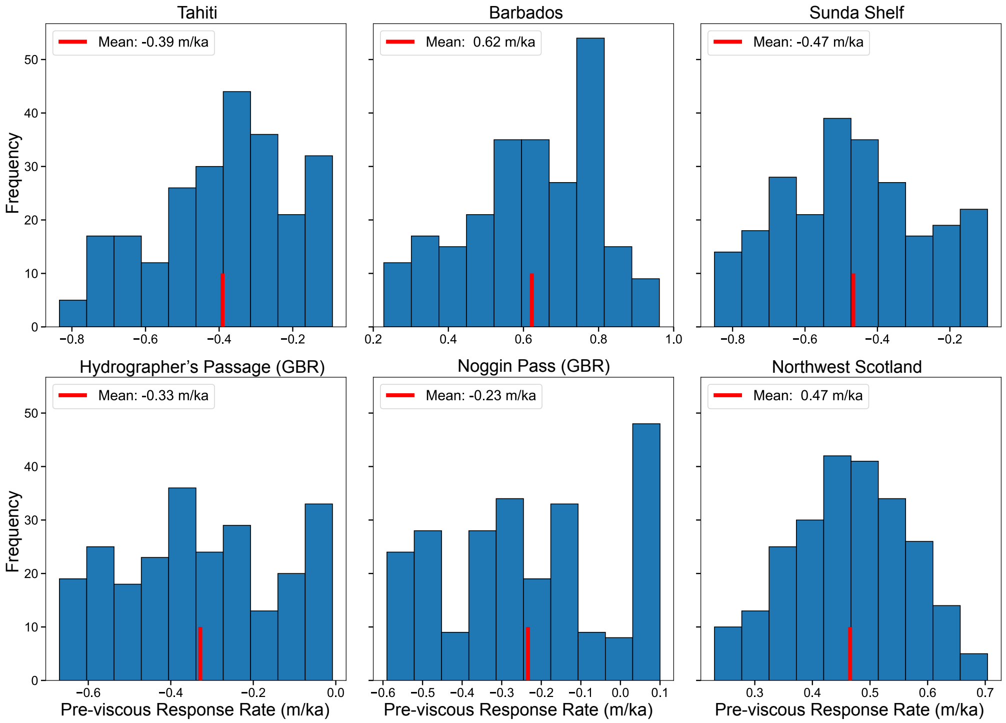

Supplementary Fig. 3: Distribution of the pre-MWP-1A viscous contribution to sea-level change at our six sites based on 240 GIA model parameter combinations. GBR = Great Barrier Reef.

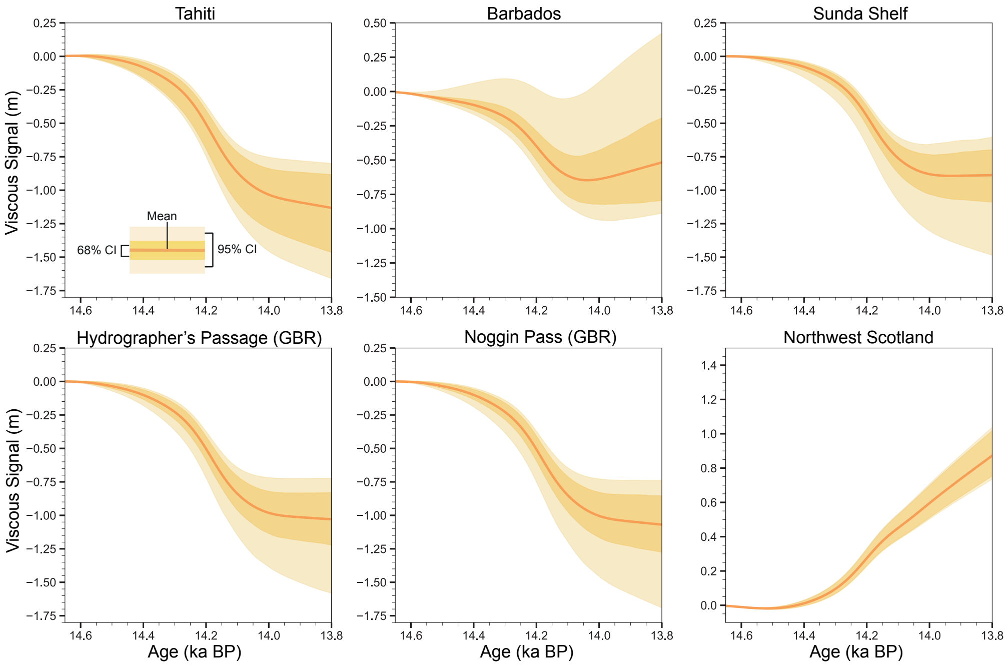

Supplementary Fig. 4: The viscous signal at our six sites due to far-field ice melt during MWP-1A. The results shown here are derived by combining 240 Earth models with the MWP-1A ice melt geometries determined by the ensemble mean of our final iteration: 13.1 m NAIS, 1.5 m AIS, and 3.3 m SIS (see Methods). CI = confidence interval.

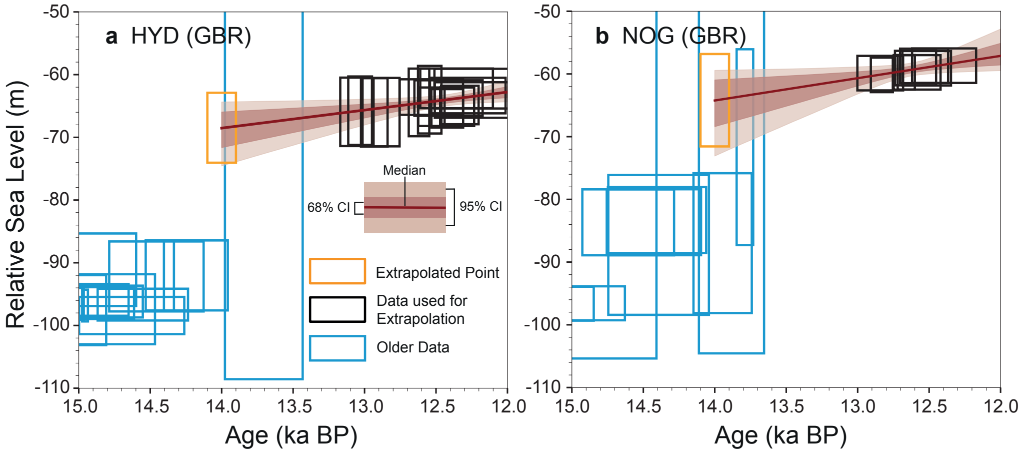

Supplementary Fig. 5: Extrapolation of relative sea-level at (a) Hydrographer's Passage and (b) Noggin Pass. Extrapolation is implemented using Monte Carlo linear regression and the uniform scenario for coral-based index points. The sea-level index points are plotted as error boxes indicating the depth range and 2σ age uncertainty. The orange square denotes the extrapolated 2σ depth and age uncertainty.

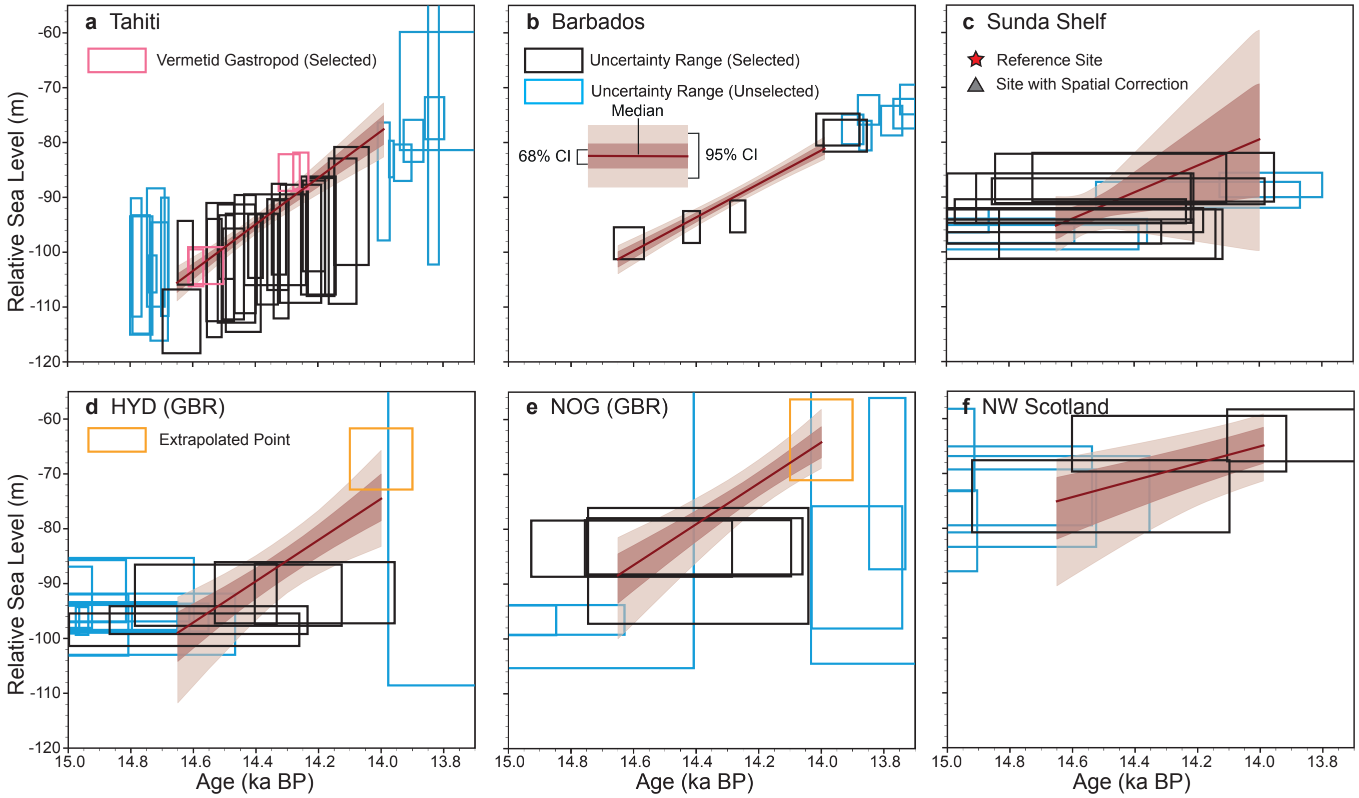

Supplementary Fig. 6: Estimated local MWP-1A sea-level rise trend at six selected sites using our uniform scenario. Same as Fig. 2 in main text but using the uniform distribution scenario for coral sea-level indicators.

Supplementary Note 1 Local GIA Signal in Northwest Scotland

The effects of the local GIA signal caused by variations of the British-Irish Ice Sheet (BIIS) are shown in Supplementary Fig. 7. We computed the likely range of the local GIA signal by combining three BIIS ice history models with 120 Earth models (Supplementary Fig. 7d-f). By subtracting the mean of the GIA ensemble from the original RSL reconstructions (blue error bars), the non-local ice-sheet induced sea-level rise signal in Northwest Scotland can be obtained (black error bars). These corrected RSL reconstructions are input into the Monte Carlo linear regression and used to estimate the local MWP-1A magnitude (Supplementary Fig. 7g-i). There are clear differences between the GIA signals associated with the three BIIS models (up to 15 m) due to differences in local ice thickness and timing of deglaciation (see original publications[1–5] for details). However, these differences do not impact on our estimate of the local MWP-1A magnitude because this only depends on the gradient of the local GIA signal between the initiation and termination of MWP-1A, which is similar for all three ice models. Consequently, the three ice models result in similar values for the local MWP-1A magnitude (Supplementary Fig. 7g-i).

Figs. 7g-i show the distributions of the local MWP-1A magnitude estimations produced by carrying out 3,000 Monte Carlo linear regression iterations for each of ice/Earth model combination (white histograms). For each ice model, the distribution of the median result for each Earth model is shown in blue. The uncertainty associated with the choice of Earth model (~0.5 m) is much smaller than that associated with the choice of ice model, which contributes to up to ~2 m difference.

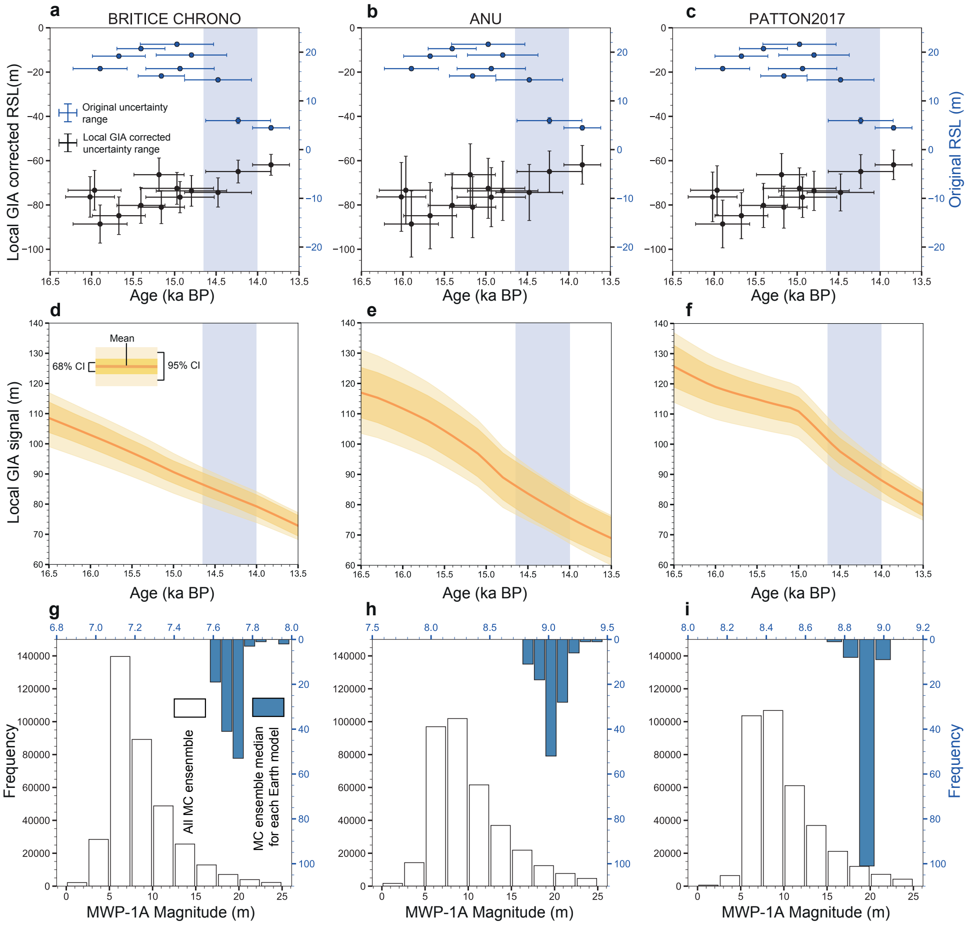

Supplementary Fig. 7: Local GIA signal correction and MWP-1A magnitude estimates using three different British-Irish Ice Sheet reconstructions[2,4,5]. (a-c) Blue error bars represent the original RSL reconstructions from sites Applecross and Arisaig[6] with 2σ age and depth uncertainties, corresponding to the blue axis shown on the right side of the figure. Black error bars represent the local GIA-corrected RSL reconstructions, with vertical error bars incorporating the 2σ uncertainty associated with 120 Earth models (index points from Arisaig have been corrected for spatial sea-level gradient), corresponding to the black axis. (d-f) Uncertainty range of the local GIA signal at Arisaig, determined using 120 Earth models. (g-i) Histograms of local MWP-1A magnitude estimates derived by combining 120 Earth models with each of the three BIIS models. After applying the local GIA signal correction associated with each Earth model, 3,000 iterations of Monte Carlo (MC) linear regression are applied to obtain ensembles of the local MWP-1A magnitude assuming a 500-year duration. A total of 360,000 outputs are shown in each the white histogram (left, black axis). For each Earth model, we calculate the median value of the associated 3,000-member ensemble and combine the results in the blue histograms (right, blue axis).

Supplementary Note 2 Jackknife Resampling - Robustness of Results

The degree to which our inversion results depend on the choice of RSL sites is assessed by jackknife resampling, which also reveal each site's contribution to the final inversion result. The inversion is repeated six times, each time removing one site from the six-site database. The results (shown in Supplementary Fig. 8) are generally consistent, pointing to a dominant NAIS contribution, except for the result obtained by excluding Barbados (third panel in Supplementary Fig. 8). When Barbados is excluded, Northwest Scotland becomes the only site that is sensitive to the source of meltwater (all other far-field sites are insensitive; see main text Fig. 1). Under this circumstance, due to the proximity of Northwest Scotland to the SIS, the Northwest Scotland MWP-1A magnitude predominantly determines the SIS inversion with the contributions from the NAIS and AIS essentially unconstrained, resulting in large uncertainty ranges (third panel in Supplementary Fig. 8). Another important feature is that excluding Northwest Scotland causes the melt contribution from the NAIS to become very large and the contribution from the SIS to become very small. The reason for this is similar, excluding Northwest Scotland leaves Barbados as the only site that is sensitive to the source of meltwater. Since Barbados is mainly sensitive to sources from the NAIS and AIS (main text Fig. 1), it is possible to partition the contribution from the NAIS and AIS, but not from the SIS. These two features are also reflected in the feasible distributions of our final inversion results shown in Supplementary Fig. 9 (same distribution as shown in main text Fig. 5 but different data representation). It is clear that the NAIS and AIS contributions to MWP-1A are strongly negatively correlated (R = -0.96), reflecting the Barbados sensitivity to the contributions from these two ice sheets. In contrast, the SIS contribution shows a weak correlation to the NAIS and AIS contributions (R = -0.30 and 0.28), indicating that the inverted SIS contribution is primarily dependent on the isolation basin stratigraphy evidence from Northwest Scotland.

We seek to resolve the ambiguities indicated by the above jackknifing process by re-running the jackknife method but excluding the SIS fingerprint, to allow us to just identify the partitioning of the NAIS and AIS contributions, which were mostly debated to be the dominant source of MWP-1A[7–12]. The results of this test are shown in Table 1, which indicates a stable solution with a dominant NAIS source (~15.5 m) and a small Antarctic contribution (~2 m). This result provides robust evidence for a NAIS dominant scenario rather than an AIS dominant scenario. However, to obtain a similarly stable jackknife result when inverting sources from three ice sheets, at least one more sea-level data site at a location that is sensitive to the MWP-1A sources is needed.

Sea-level index points from the Argentine Shelf could provide a powerful constraint since local RSL variation will be sensitive to melt from the nearby AIS. However, although shell sediment records from the Argentine Shelf have been dated through the MWP-1A interval[13], the indicative meaning of shell sea-level indicators remains problematic (see Guilderson et al.[13]). Additionally, RSL change on the Argentine Shelf will be influenced by variations in the adjacent Patagonian Ice Sheet, the deglaciation history of which, though improved by a recent glacial geomorphology data compilation of Davies et al.[14], has not yet been calibrated with sea-level data. Sea-level index points from locations such as Bonaparte Gulf[15,16] and Echigo Plain, Japan[17] are also dated to across MWP-1A, but due to the lack of temporal resolution and their insensitivity to the sources of MWP-1A, they are not particularly useful for our fingerprinting technique. Lastly, using the local GIA correction method introduced in this study, it would be possible to use other near-field sea-level constraints for MWP-1A source inversion (e.g., records from southwest Norway[18]). However, estimating the local GIA signal requires a well-studied local deglaciation history and sufficient temporal resolution during MWP-1A, which currently is only achieved in Northwest Scotland.

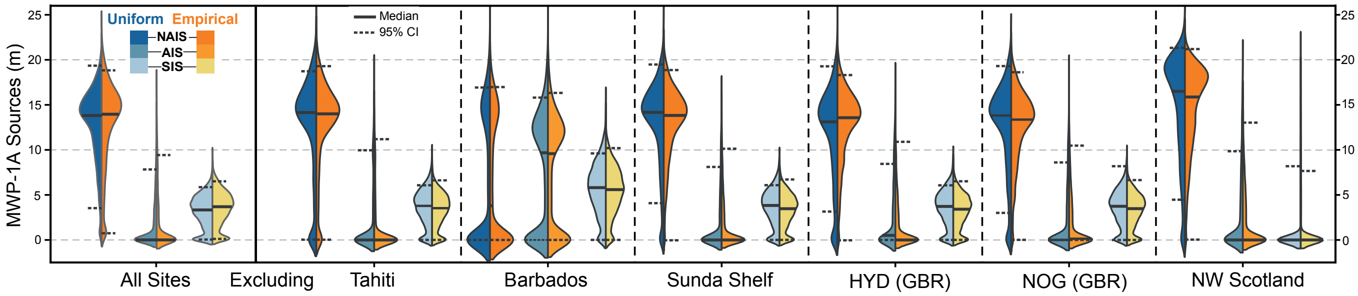

Supplementary Fig. 8: A violin plot showing the jackknife resampling results. The first panel shows the MWP-1A source inversion results obtained using all six sites in the database, and the second to seventh panel show the results when excluding each named site from the six-site database. Each panel consists of three violin plots, corresponding to the contribution from each ice sheet: NAIS, AIS, and SIS. The blue/orange tone of the violin plot indicates the result generated using the uniform/empirical scenario for coral records.

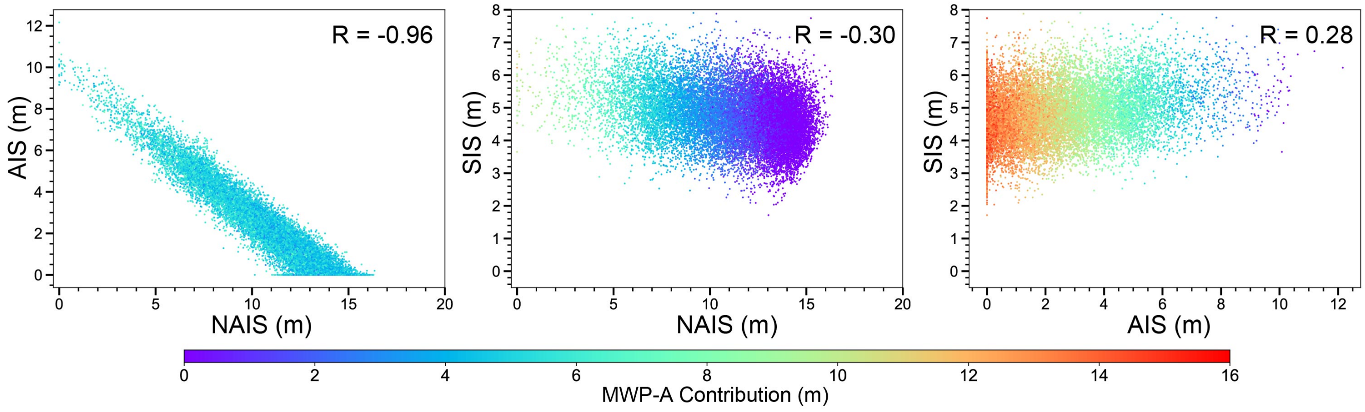

Supplementary Fig. 9: Feasible distributions of sea-level oscillation limit constrained inversion result. This is an alternative representation of the information shown in Fig. 5 of the main text. Each scatter point represents an inversion result from one of 20,000 MC simulations. In each plot, the MWP-1A contribution of the ice sheet that is not listed on the x and y axes is represented by the colour of the dots. The correlation coefficient of each scatter plot is listed within the plot.

Excluded Site	NAIS (m)	AIS (m)
Tahiti	15.48 [10.10-19.61]	2.16 [0-6.13]
Barbados	15.98 [11.52-19.40]	1.26 [0.00-0.31]
Sunda Shelf	15.84 [11.52-19.40]	2.03 [0.00-5.25]
Hydrographer's Passage (GBR)	14.68 [9.24-18.92]	2.64 [0.00-6.74]
Noggin Pass (GBR)	15.21 [10.42-19.18]	2.34 [0.00-6.01]
NW Scotland	14.36 [7.61-19.73]	3.83 [0.00-9.10]
Bias-Contained Result	15.57 [11.03-19.28]	2.16 [0.00-5.63]
Jackknife Average	15.26 [11.12-19.05]	2.38 [0.00-4.83]
Bias-Corrected Result	15.89 [11.38-19.60]	1.95 [0.00-5.41]

Supplementary Table 1: Jackknife resampling results when excluding the Scandinavian Ice Sheet contribution. The results are based on the empirical distribution scenario for coral-based index points. Rows with site location shown on the left document the inversion results when that site is removed from the six-site database. The last three rows show the bias-contained inversion result, the averaged results of the six jackknife tests, and the bias-corrected result, respectively. The first value in each box is the ensemble mean value of 20,000 iterations of the inversion process, the values in square brackets are the 68% confidence interval.

Supplementary Note 3 Isolation Basin Evidence

This section provides detailed interpretations of Scottish isolation basin stratigraphy to support the definition of the sea-level oscillation limit used in the main text.

In addition to providing sea-level index points, the isolation basin records from Northwest Scotland provide a unique insight into the magnitude and sources of MWP-1A due to their near-field location and the wealth of information contained within the sedimentation staircase[19]. Prior to MWP-1A, the dominant signal recorded by Northwest Scotland sea-level index points is uplift-induced sea-level fall triggered by local ice loss. With this continuous sea-level fall, some basins that were originally connected with the ocean would become isolated. This process is represented by a sediment phase transition from a marine phase (silt/clay) to a freshwater phase (organic mud and peat, Supplementary Fig. 10a). During MWP-1A, if the rate of sea-level rise due to melt from the NAIS, AIS and SIS outpaced the local rate of land uplift, it would case isolation basins in the right height window to reconnect to the ocean, resulting in two isolation events (i.e., marine-freshwater-marine-freshwater, so-called sea-level oscillation), which should be recorded by sediment stratigraphy (Supplementary Fig. 10b). However, for all isolation basins at four sites across Northwest Scotland (Applecross, Arisaig, Kentra and Kintyre; main text Fig. 4g) that were isolated shortly before or during MWP-1A, there is no sea-level oscillation recorded (see isolation basin stratigraphic reconstructions in Shennan et al.[20–23]). These records therefore provide strong evidence that during MWP-1A the rate of sea-level rise in Northwest Scotland due to far-field ice melt cannot have significantly outpaced the local land uplift rate (i.e., there was no or only a very minor RSL oscillation[19,22–25]).

This condition is met by our MWP-1A source partition, the RSL predictions generated by the ANU_MWP ice model (Supplementary fig. 11a-d) show a monotonic sea-level fall across MWP-1A and provide good fit to SLIPs at different periods for Arisaig, Kentra and Kintyre. The only minor sea-level oscillation occurs at Applecross where local RSL is underestimated, indicating the adopted ice thickness in this region is too thin in the ANU_MWP ice model.

We place an upper bound on the rate of land uplift (i.e., sea level oscillation limit) at Arisaig during MWP-1A by identifying the largest rate predicted by combining the ANU and BRITICE-CHRONO ice models with 120 Earth models that closely reflect mantle properties beneath the British Isles[26]. The maximum rate produces 9.8 m uplift in 500 years obtained by combining the ANU model with the weakest upper and lower mantle model of $4/100 \times 10^{20}$ Pa s, respectively. The PATTON2017 model may produce a larger land uplift rate, but it has not been calibrated to any sea-level records across Scotland and therefore the resulting land uplift rate is less meaningful as a constraint for MWP-1A magnitude.

Several previous studies have used isolation basin evidence to constrain the MWP-1A magnitude and sources[6,21,23,27]. When a regional BIIS model is combined with a global ice model (e.g., a revised version of the model of Bassett et al.[9]) with a dominant AIS contribution to MWP-1A[28,29], this results in a strong sea-level oscillation across Scotland during MWP-1A (see Fig. 10 of Shennan et al.[6]). Because the idea of a substantial SIS contribution has only recently been proposed, previous studies inferred a lower global MWP-1A magnitude to avoid this RSL oscillation, but this is inconsistent with our estimations 20 m sea-level rise in the far field. One way to improve this inconsistency is to estimate MWP-1A magnitude and sources using far-field and near-field data together. Liu[27] provides a novel method that combines isolation basin evidence with far-field sea-level records from Tahiti, Barbados and Sunda Shelf to infer the MWP-1A sources from the NAIS and AIS. However, the method adopted to remove the local GIA signal for Scotland in that study results in significant uncertainty, which makes the sea-level index points from Scotland less useful for inverting the MWP-1A sources. Liu[27] therefore was not able to exclusively rule out either a small or large Antarctic contribution.

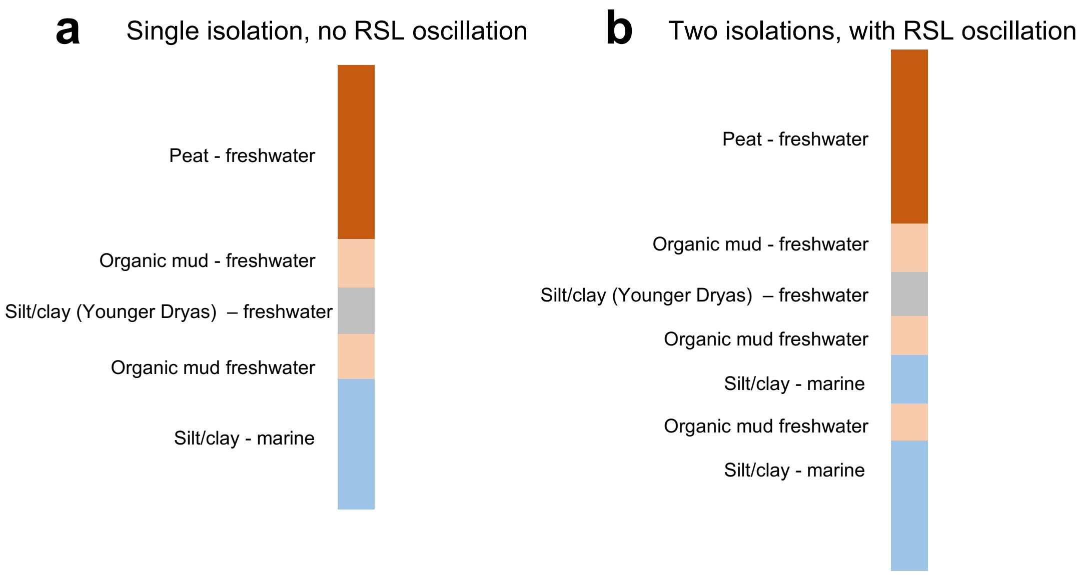

Supplementary Fig. 10: Two stratigraphic models of the isolation process. (a) A single isolation process model, indicating no RSL oscillation across MWP-1A; (b) A double isolation process model, indicating an RSL oscillation across MWP-1A.

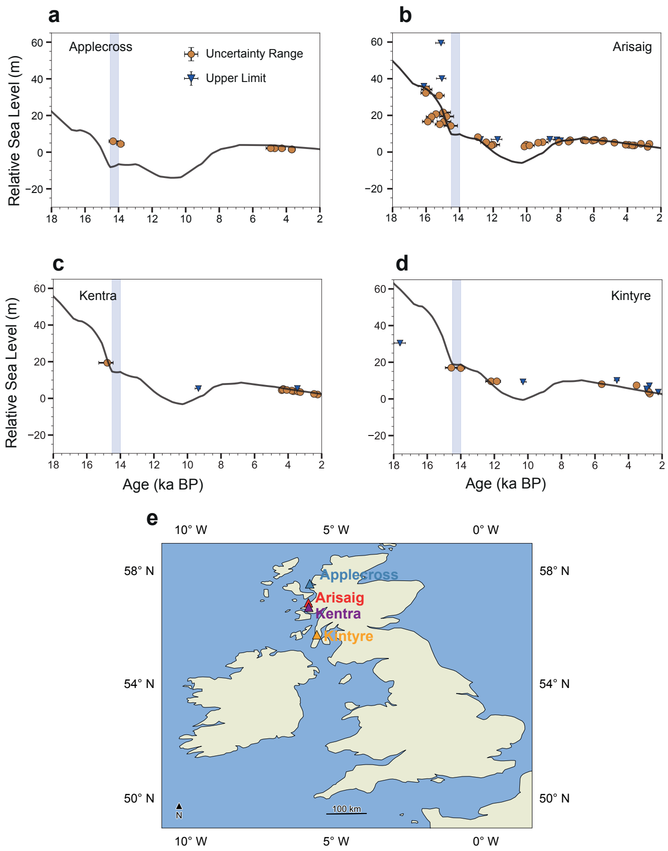

Supplementary Fig. 11: Predicted relative sea-level across Northwest Scotland. (a-d) Relative sea-level predictions generated using the ANU_MWP model (with optimum Earth model of 65 km lithospheric thickness, 4/200 $\times$ 10^{20} Pa s upper/lower mantle viscosity) at Applecross, Arisaig, Kentra and Kintyre, respectively. Error bars show 2σ depth and age uncertainties. (e) Locations of four Scottish sites, Applecross (blue), Arisaig (red), Kentra (purple) and Kintyre (orange).

Ice Sheet	Method	Duration (yrs)	MWP-1A Contribution (m)	Reference
NAIS	Data-driven inversion with GIA modelling	500	5.6-15.4	this study
NAIS	Ice area-volume transition	800	6.7 – 8.7 (SLE)	Carlson et al.[30]
NAIS	GIA analysis (ICE-5G)	500	20-20.5	Peltier et al.[8]
NAIS	GIA analysis (ICE-6G_C)	500	16.5-18	Peltier et al.[31]
NAIS	3D glacial systems model	500	7.7-10.2	Tarasov et al.[32]
NAIS	Revisited glacial systems model with Bayesian style calibration	500	9.4-13.2	Tarasov et al.[12]
NAIS	Saddle collapse ice dynamic	340 (500)	5.7-11 (10.4)	Gregoire et al.[11,33]; Gomez et al.[34]
NAIS	GIA and tilting of glacial lake shoreline analysis	600	~ 18.7	Lambeck et al.[35]
AIS	Data-driven inversion with GIA modelling	500	0-5.9	this study
AIS	GIA analysis (ICE6G_C)	500	1.5-2	Argus et al.[36]
AIS	Global sea-level budget analysis	500	~0.5	Lambeck et al.[2]
AIS	Ice sheet dynamic modelling	500	0.5/1.5 (W12/W12 S2)	Whitehouse et al.[37,38]
AIS	Ice sheet dynamic modelling forced by output from an earth system modelling	500	Up to 2	Golledge et al.[39]
AIS	Coupled ice-sheet and sea-level modelling	340	1.0-2.0 (SLE)	Gomez et al.[40]
AIS	GIA analysis of far-field sea-level data within a Bayesian framework	340	Either 4.1-10 or 0-6.9	Liu et al.[41]
AIS	GIA analysis of far-/near-field sea-level data within a Bayesian framework	340	Either 5-10 or 2-7	Liu[27]
AIS	GIA analysis of far-field sea-level data	340	At least 7	Deshamps et al.[10]
AIS	GIA analysis of far-field sea-level data	1000	Preferably 15	Bassett et al.[9]
SIS	Data-driven inversion with GIA modelling	500	3.2-6.4	this study
SIS	Thermomechanical ice modelling	340	2.5 (SLE)	Patton et al.[3,4]
SIS	GIA analysis (ICE-6G_C)	500	3.5-4	Peltier et al.[31]
SIS	GIA analysis	500	0.8-1	Lambeck et al.[2]
SIS	Compilation of large geomorphological dataset; ice area-volume transition	500	1.7-2	Hughes et al.[42]
SIS	Chronological reinterpretation of SIS ice sheet margin; ice area-volume transition	500	4.5-7.9 (SLE)	Brendryen et al.[43]
SIS	Ice area-volume transition	1000	4.1-5.7 (SLE)	Carlson et al.[30]

Supplementary Table 2: Estimates of the contribution of each ice sheet to MWP-1A. NAIS = North American Ice Sheet; AIS = Antarctic Ice Sheet; SIS = Scandinavian & Barents Sea Ice Sheet. Estimates derived using the ice area-volume transition method are presented in sea-level equivalent (SLE), which includes the contribution from ice that lies below hydrostatic equilibrium (which will not contribute to global sea-level rise); other estimates reflect the eustatic sea level contribution.

Ice Sheet	NAIS[a]	NAIS	NAIS	NAIS	WLIS	ELIS	SIS[a]	SIS	SIS[b]
Ice Model	ANU[35]	ICE6G_C[31]	GLAC_1D[12]	G12[11,34]	ICE6G_C[31]		BRITICE CHRONO[5]	PATTON2017[4]	PATTON2017[4]
**Sea-level fingerprint value**									
Tahiti	1.21	1.21	1.22	1.24[c]	1.27	1.19	0.98	0.98	0.99
Barbados	0.82	0.79	0.81	0.85[c]	0.96	0.69	0.97	0.97	0.97
Sunda Shelf	1.08	1.08	1.09	1.09[c]	1.11	1.08	1.00	0.99	1.00
HYD (GBR)	1.04	1.03	1.04	1.07[c]	1.12	1.00	0.97	0.97	0.98
NOG (GBR)	1.03	1.02	1.03	1.06[c]	1.10	0.99	0.96	0.97	0.98
NW Scotland	0.75	0.75	0.79	0.82[c]	0.93	0.64	-0.74	-0.76	-0.60
**Inversion results**									
Total (m)	17.9 [15.6-20.3]	18.1 [15.7-20.6]	17.7 [15.6-20.3]	17.6 [15.4-19.4]	18.1 [15.3-21.3]		17.9 [15.6-20.3]	17.9 [15.6-20.3]	17.8 [15.6-20.3]
NAIS (m)	13.1 [6.0-18.3]	12.9 [5.3-18.0]	12.7 [5.6-17.8]	12.7 [5.8-17.4]	4.4 [0-12.5]	8.7 [0-16.8]	13.1 [6.0-18.3]	13.1 [6.0-18.3]	12.8 [6.0-18.1]
AIS (m)	1.5 [0-6.9]	1.7 [0-7.2]	1.5 [0-6.9]	1.2 [0-6.6]	1.5 [0-6.9]		1.5 [0-6.9]	1.5 [0-6.9]	1.4 [0-6.8]
SIS (m)	3.3 [0.5-6.0]	3.5 [0.7-6.3]	3.7 [0.7-6.4]	3.7 [0.7-6.3]	3.5 [0.4-6.1]		3.3 [0.5-6.0]	3.3 [0.4-5.9]	3.6 [0.5-6.6]

Supplementary Table 3: Sea-level fingerprint values and inversion results associated with using different ice melt geometries. The inversion results shown here have not been constrained by the sea-level oscillation limit. For the inversion results, the first value in each box is the ensemble mean value of 20,000 iterations of the inversion process, the values in square brackets are the 95% confidence interval. WLIS = West Laurentide Ice Sheet, ELIS = East Laurentide Ice Sheet, separated by 110° W. (a) Ice melt geometries used in our inversion. (b) SIS melt geometry associated with Southern Barents Sea sector collapse. (c) Sea-level fingerprints were estimated from Gomez et al.[34] in their Fig. 2.

Supplementary References

1. Lambeck, K. Glacial rebound of the British Isles—II. A high-resolution, high-precision model. *Geophysical Journal International* **115,** 960–990 (1993).
2. Lambeck, K., Rouby, H., Purcell, A., Sun, Y. & Sambridge, M. Sea level and global ice volumes from the Last Glacial Maximum to the Holocene. *Proceedings of the National Academy of Sciences* **111,** 15296–15303 (2014).
3. Patton, H., Hubbard, A., Andreassen, K., Winsborrow, M. & Stroeven, A. P. The build-up, configuration, and dynamical sensitivity of the Eurasian ice-sheet complex to Late Weichselian climatic and oceanic forcing. *Quaternary Science Reviews* **153,** 97–121 (2016).
4. Patton, H. *et al.* Deglaciation of the Eurasian ice sheet complex. *Quaternary Science Reviews* **169,** 148–172 (2017).
5. Clark, C. D. *et al.* BRITICE Glacial Map, version 2: a map and GIS database of glacial landforms of the last British–Irish Ice Sheet. *Boreas* **47,** 11–27 (2018).
6. Shennan, I., Bradley, S. L. & Edwards, R. Relative sea-level changes and crustal movements in Britain and Ireland since the Last Glacial Maximum. *Quaternary Science Reviews* **188,** 143–159 (2018).
7. Weaver, A. J., Saenko, O. A., Clark, P. U. & Mitrovica, J. X. Meltwater pulse 1A from Antarctica as a trigger of the Bølling-Allerød warm interval. *Science* **299,** 1709–1713 (2003).
8. Peltier, W. R. Global glacial isostasy and the surface of the ice-age Earth: the ICE-5G (VM2) model and GRACE. *Annu. Rev. Earth Planet. Sci.* **32,** 111–149 (2004).
9. Bassett, S. E., Milne, G. A., Mitrovica, J. X. & Clark, P. U. Ice sheet and solid earth influences on far-field sea-level histories. *Science* **309,** 925–928 (2005).
10. Deschamps, P. *et al.* Ice-sheet collapse and sea-level rise at the Bølling warming 14,600 years ago. *Nature* **483,** 559–564 (2012).
11. Gregoire, L. J., Payne, A. J. & Valdes, P. J. Deglacial rapid sea level rises caused by ice-sheet saddle collapses. *Nature* **487,** 219–222 (2012).
12. Tarasov, L., Dyke, A. S., Neal, R. M. & Peltier, W. R. A data-calibrated distribution of deglacial chronologies for the North American ice complex from glaciological modeling. *Earth and Planetary Science Letters* **315,** 30–40 (2012).
13. Guilderson, T., Burckle, L., Hemming, S. & Peltier, W. Late Pleistocene sea level variations derived from the Argentine Shelf. *Geochemistry, Geophysics, Geosystems* **1** (2000).
14. Davies, B. J. *et al.* The evolution of the Patagonian Ice Sheet from 35 ka to the present day (PATICE). *Earth-Science Reviews,* 103152 (2020).
15. Yokoyama, Y., Lambeck, K., De Deckker, P., Johnston, P. & Fifield, L. K. Timing of the Last Glacial Maximum from observed sea-level minima. *Nature* **406,** 713–716 (2000).
16. Ishiwa, T. *et al.* A sea-level plateau preceding the Marine Isotope Stage 2 minima revealed by Australian sediments. *Scientific reports* **9,** 1–8 (2019).
17. Tanabe, S., Tateishi, M. & Shibata, Y. The sea-level record of the last deglacial in the Shinano River incised-valley fill, Echigo Plain, central Japan. *Marine Geology* **266,** 223–231 (2009).
18. Vasskog, K. *et al.* Evidence of early deglaciation (18 000 cal a bp) and a postglacial relative sea-level curve from southern Karmøy, south-west Norway. *Journal of Quaternary Science* **34,** 410–423 (2019).

19. Shennan, I. *et al.* Late Devensian and Holocene records of relative sea-level changes in northwest Scotland and their implications for glacio-hydro-isostatic modelling. *Quaternary Science Reviews* **19,** 1103–1135 (2000).
20. Shennan, I., Innes, J. B., Long, A. J. & Zong, Y. Late Devensian and Holocene relative sea-level changes at Rumach, near Arisaig, northwest Scotland. *Norsk Geologisk Tidsskrift* **73,** 161–174 (1993).
21. Shennan, I. *et al.* Evaluation of rapid relative sea-level changes in north-west Scotland during the last Glacial-Interglacial transition: evidence from Ardtoe and other isolation basins. *Journal of Coastal Research,* 862–874 (1996).
22. Shennan, I., Rutherford, M. M., Innes, J. B. & Walker, K. J. Late glacial sea level and ocean margin environmental changes interpreted from biostratigraphic and lithostratigraphic studies of isolation basins in northwest Scotland. *Geological Society, London, Special Publications* **111,** 229–244 (1996).
23. Shennan, I. *et al.* Relative sea-level changes, glacial isostatic modelling and ice-sheet reconstructions from the British Isles since the Last Glacial Maximum. *Journal of Quaternary Science: Published for the Quaternary Research Association* **21,** 585–599 (2006).
24. Shennan, I., Innes, J. B., Long, A. J. & Zong, Y. Late Devensian and Holocene relative sealevel changes at Loch nan Eala, near Arisaig, northwest Scotland. *Journal of Quaternary Science* **9,** 261–283 (1994).
25. Shennan, I. Global meltwater discharge and the deglacial sea-level record from northwest Scotland. *Journal of Quaternary Science* **14,** 715–719 (1999).
26. Bradley, S., Milne, G., Teferle, F. N., Bingley, R. & Orliac, E. Glacial isostatic adjustment of the British Isles: new constraints from GPS measurements of crustal motion. *Geophysical Journal International* **178,** 14–22 (2009).
27. Liu, J. *Constraining the Source Distribution of Meltwater Pulse 1A Using Near-and Far-Field Sea-level Data.* MA thesis (University of Ottawa, 2013).
28. Bradley, S. L., Milne, G. A., Shennan, I. & Edwards, R. An improved glacial isostatic adjustment model for the British Isles. *Journal of Quaternary Science* **26,** 541–552 (2011).
29. Kuchar, J. *et al.* Evaluation of a numerical model of the British–Irish ice sheet using relative sea-level data: implications for the interpretation of trimline observations. *Journal of Quaternary Science* **27,** 597–605 (2012).
30. Carlson, A. E. & Clark, P. U. Ice sheet sources of sea level rise and freshwater discharge during the last deglaciation. *Reviews of Geophysics* **50** (2012).
31. Peltier, W., Argus, D. & Drummond, R. Space geodesy constrains ice age terminal deglaciation: The global ICE-6G_C (VM5a) model. *Journal of Geophysical Research: Solid Earth* **120,** 450–487 (2015).
32. Tarasov, L. & Peltier, W. A calibrated deglacial drainage chronology for the North American continent: evidence of an Arctic trigger for the Younger Dryas. *Quaternary Science Reviews* **25,** 659–688 (2006).
33. Gregoire, L. J., Otto-Bliesner, B., Valdes, P. J. & Ivanovic, R. Abrupt Bølling warming and ice saddle collapse contributions to the Meltwater Pulse 1a rapid sea level rise. *Geophysical research letters* **43,** 9130–9137 (2016).
34. Gomez, N., Gregoire, L., Mitrovica, J. & Payne, A. Laurentide-Cordilleran Ice Sheet saddle collapse as a contribution to meltwater pulse 1A. *Geophysical Research Letters* **42,** 3954–3962 (2015).
35. Lambeck, K., Purcell, A. & Zhao, S. The North American Late Wisconsin ice sheet and mantle viscosity from glacial rebound analyses. *Quaternary Science Reviews* **158,** 172–210 (2017).
36. Argus, D. F., Peltier, W., Drummond, R. & Moore, A. W. The Antarctica component of postglacial rebound model ICE-6G_C (VM5a) based on GPS positioning, exposure age dating of ice thicknesses, and relative sea level histories. *Geophysical Journal International* **198,** 537–563 (2014).
37. Whitehouse, P. L., Bentley, M. J. & Le Brocq, A. M. A deglacial model for Antarctica: geological constraints and glaciological modelling as a basis for a new model of Antarctic glacial isostatic adjustment. *Quaternary Science Reviews* **32,** 1–24 (2012).
38. Whitehouse, P. L., Bentley, M. J., Milne, G. A., King, M. A. & Thomas, I. D. A new glacial isostatic adjustment model for Antarctica: calibrated and tested using observations of relative sea-level change and present-day uplift rates. *Geophysical Journal International* **190,** 1464–1482 (2012).
39. Golledge, N. *et al.* Antarctic contribution to meltwater pulse 1A from reduced Southern Ocean overturning. *Nature communications* **5,** 5107 (2014).
40. Gomez, N., Weber, M. E., Clark, P. U., Mitrovica, J. X. & Han, H. K. Antarctic ice dynamics amplified by Northern Hemisphere sea-level forcing. *Nature* **587,** 600–604 (2020).
41. Liu, J., Milne, G. A., Kopp, R. E., Clark, P. U. & Shennan, I. Sea-level constraints on the amplitude and source distribution of Meltwater Pulse 1A. *Nature Geoscience* **9,** 130–134 (2016).
42. Hughes, A. L., Gyllencreutz, R., Lohne, Ø. S., Mangerud, J. & Svendsen, J. I. The last Eurasian ice sheets–a chronological database and time-slice reconstruction, DATED-1. *Boreas* **45,** 1–45 (2016).
43. Brendryen, J., Haflidason, H., Yokoyama, Y., Haaga, K. A. & Hannisdal, B. Eurasian Ice Sheet collapse was a major source of Meltwater Pulse 1A 14,600 years ago. *Nature Geoscience* **13,** 363–368 (2020).
